# Deep Learning Network Selection and Optimized Information Fusion for Enhanced COVID-19 Detection: A Literature Review

**DOI:** 10.3390/diagnostics15141830

**Published:** 2025-07-21

**Authors:** Olga Adriana Caliman Sturdza, Florin Filip, Monica Terteliu Baitan, Mihai Dimian

**Affiliations:** 1 Faculty of Medicine and Biological Sciences, Stefan cel Mare University of Suceava, 720229 Suceava, Romania; florin.filip@usm.ro (F.F.); monicst@yahoo.com (M.T.B.); 2 Emergency Clinical Hospital Suceava, 720237 Suceava, Romania; 3 Integrated Center for Research, Development and Innovation in Advanced Materials, Nanotechnologies, and Distributed Systems for Fabrication and Control, Stefan cel Mare University of Suceava, 720229 Suceava, Romania; dimian@usm.ro; 4 Department of Computers, Electronics and Automation, Stefan cel Mare University of Suceava, 720229 Suceava, Romania

**Keywords:** artificial intelligence, convolutional neural networks, vision transformers, chest X-ray, computer tomography, diagnosis, image classification, medical image analysis

## Abstract

The rapid spread of COVID-19 increased the need for speedy diagnostic tools, which led scientists to conduct extensive research on deep learning (DL) applications that use chest imaging, such as chest X-ray (CXR) and computed tomography (CT). This review examines the development and performance of DL architectures, notably convolutional neural networks (CNNs) and emerging vision transformers (ViTs), in identifying COVID-19-related lung abnormalities. Individual ResNet architectures, along with CNN models, demonstrate strong diagnostic performance through the transfer protocol; however, ViTs provide better performance, with improved readability and reduced data requirements. Multimodal diagnostic systems now incorporate alternative methods, in addition to imaging, which use lung ultrasounds, clinical data, and cough sound evaluation. Information fusion techniques, which operate at the data, feature, and decision levels, enhance diagnostic performance. However, progress in COVID-19 detection is hindered by ongoing issues stemming from restricted and non-uniform datasets, as well as domain differences in image standards and complications with both diagnostic overfitting and poor generalization capabilities. Recent developments in COVID-19 diagnosis involve constructing expansive multi-noise information sets while creating clinical process-oriented AI algorithms and implementing distributed learning protocols for securing information security and system stability. While deep learning-based COVID-19 detection systems show strong potential for clinical application, broader validation, regulatory approvals, and continuous adaptation remain essential for their successful deployment and for preparing future pandemic response strategies.

## 1. Introduction

The COVID-19 pandemic has created an urgent need for rapid and accurate diagnostic tools. While the gold-standard RT-PCR test is specific, it can suffer from sensitivity issues and delayed results. Scientists are examining deep learning (DL) models for analyzing medical data, primarily chest imaging, to provide fast and accurate detection of COVID-19 pneumonia [1]. The characteristic lung manifestations of COVID-19 in patients, such as ground-glass opacities, can be identified through chest radiography (X-ray) and computed tomography scans, thus making these methods suitable for artificial intelligence (AI)-based screening applications [2]. Convolutional neural networks (CNNs) are part of deep neural networks, which excel at pattern recognition in images and sometimes surpass expert radiological evaluation of these images [3]. This review examines how deep learning architectures detect COVID-19, the data modalities—X-ray and CT—used, and information fusion techniques that enhance diagnostic precision. This study analyzes technological methods and assesses model performance and evaluation metrics from different studies while discussing the main difficulties and the latest advancements (2020–2024) relevant to this domain. The data foundation for this narrative review comprises various peer-reviewed research and survey materials from 2020 to 2024, including significant scholarly publications and combined observations from publicly available datasets. Cited references appear throughout the text to verify both the results and claims presented in the summary. The included tables present consolidated results from survey papers, representative reports, and individual studies as examples.

## 2. Methods

This narrative review aimed to synthesize the literature on novel COVID-19 diagnostic systems based on deep learning of medical CT and X-ray imaging samples. The articles reviewed were selected from the PubMed, Google Scholar, and Scopus databases. Research articles, meta-analyses, systematic reviews, narrative reviews, and some conference proceedings were included. Articles not in English were excluded, and peer-reviewed studies were preferred. The information obtained from the evaluated studies is presented in a clear, concise, and accessible language for the clinician, taking into account some important components, such as data obtained from experiments, different classification methods based on DL, and the performance evaluation metrics used. This review also highlights potential future lines of research on the development of artificial intelligence-based COVID-19 detection systems to ensure rapid and accurate diagnosis.

This study has several limitations. First, it considers a limited number of review articles, which assumes a certain level of domain-specific knowledge; for this reason, this narrative review does not cover all the details of deep machine learning and neural networks. Second, the review focuses on the contribution of artificial intelligence to the management of SARS-CoV-2 infection and does not provide technical details about neural networks.

## 3. Data Modalities for COVID-19 Diagnosis

**Figure 1:**
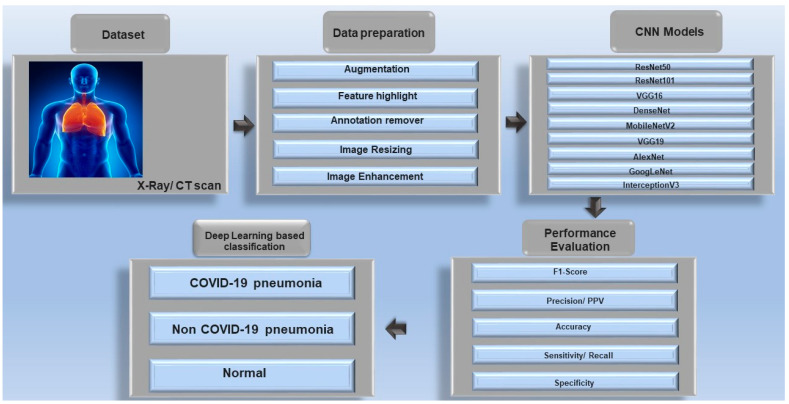
DL model for the classification of COVID-19 based on an image dataset (The arrows illustrate the sequence of steps within the workflow).

Multiple approaches based on DL have been developed for the diagnosis of COVID-19. The steps for using DL in identifying and classifying types of pneumonia involve the availability of datasets, which typically consist of CT scans or X-ray images. The data is preprocessed according to DL requirements, after which the appropriate type of CNN model is selected. Following data preparation and analysis, important information is extracted that can subsequently lead to the classification of different types of pneumonia (Figure 1).

### 3.1. Chest Radiography (X-Ray)

Chest X-rays serve as the main diagnostic imaging procedure for patients presenting with breathing problems. Large-scale COVID-19 screening benefits from their fast evaluation process, inexpensive nature, and wide availability [4]. The diagnosis of COVID-19 infection through CXRs remains challenging because the exam results appear subtle and resemble other pneumonias and different lung conditions. The figure shows typical CXR images; however, it remains very difficult to distinguish healthy lungs from infected ones through bare human observation [5]. The year 2020 saw the generation of numerous CXR image datasets and their deposition in public repositories, improving DL model capabilities [6]. COVID-19 radiographic examinations of the chest typically display patchy opacities, which may be located at the edges and both sides of the lungs; however, these presentations are also found in other pulmonary disorders. The initial artificial intelligence algorithms attempted to identify COVID-19 from normal lung scans and non-COVID-19 pneumonia findings by detecting under-recognized X-ray characteristics [7]. The reported accuracy rates for CXR-based AI systems in test datasets remain high (Table 1), as indicated by the studies presented; however, testing generalization requires more reliable training data.

### 3.2. Computed Tomography (CT)

CT chest imaging produces images showing detailed three-dimensional lung parenchyma structures while simultaneously making it possible to detect COVID-19 characteristic findings such as ground-glass opacities and consolidations [17]. Numerous DL studies analyzed the presence of COVID-19 using CT image datasets that were widespread in their regions. The primary objective of CT-based models is to distinguish COVID-19 pneumonia from other types of pneumonia and normal lung conditions [18]. Several studies have employed CT technology to predict both COVID-19 disease severity and radiological findings. CT images provide better resolution than CXR, as well as 3D analysis capabilities; however, they also introduce complexities due to their greater dimensional scope and the variations in imaging characteristics between different scanners used in healthcare centers [19]. The collection of CT imaging data in multiple hospitals has revealed the difficulty of a “domain shift” due to distinct image modules between facilities, which reduces model effectiveness [20,21]. This issue was resolved using normalization methods in conjunction with domain adaptation techniques. Research based on CNNs of CT imaging data produced excellent results (over 90% accuracy) during within-dataset evaluations (Table 2).

ResNet152 achieved 95% accuracy when used to detect COVID-19 on CT images from 1881 cases [20]. A pre-trained ResNet101 model achieved 99.5% accuracy when analyzing 1020 CT images through transfer learning techniques [30]. High evaluation results tend to stem from assessments of small, homogeneous test datasets; large-scale investigations reveal better and more conservative outcomes, with 92% accuracy and an AUC of 0.95 obtained for multi-classification of 89,000 scans [20,29].

### 3.3. Other Modalities (Ultrasound, Audio, and Clinical Data)

Researchers have also explored different forms of data beyond X-rays and CT scans. A lung ultrasound examination can reveal both pleural irregularities and B-lines that aid in detecting COVID-19 pneumonia during a patient examination. Lung ultrasound images classified using deep learning models demonstrated between 81% and 96% sensitivity and specificity for detecting COVID-19 lung patterns in medical studies [31,32]. The analysis of cough sounds represents another modality that has been used by some researchers for classifying COVID-19 through deep learning techniques, resulting in promising yet out-of-scope results [20,33]. Multiple studies in this field place strong emphasis on merging clinical information that combines patient symptoms, vital signs, laboratory testing, and imaging modalities [34,35]. Dr. Ruesholtzke et al. illustrated how blood biomarkers, such as inflammatory markers, provide information supplementary to imaging data [34]. The diagnostic accuracy of CXR images improves when doctors integrate the images with clinical laboratory data through a multi-model approach [3]. Hardy-Werbin et al. (2023) developed the multimodal system “MultiCOVID”, which combines chest X-rays with blood test results to distinguish COVID-19 from heart failure, other forms of pneumonia, and healthy conditions [3]. The developed system achieved 84% accuracy (AUC~0.92) and outperformed expert radiologists in diagnosing COVID-19 from CXR scans. The combined use of different data sources highlights their advantage for improved COVID-19 diagnosis.

## 4. Deep Learning Models and Architectures for COVID-19 Detection

### 4.1. Convolutional Neural Networks (CNNs)

Deep CNNs function as the primary method for classifying COVID-19 images [36]. A large number of deep CNN architectures, including VGG, ResNet, Inception, Xception, DenseNet, and MobileNet, were used for CXR and CT image analysis in 2020 [9,10,11,20,22,23,25,27,30,37,38,39].

Since specific datasets for COVID-19 images were lacking, researchers used ImageNet-pretrained models through transfer learning as the default approach [40]. Scientists adapted the top parts of pre-trained CNNs by replacing them to develop COVID-19 detection capabilities [41]. This method used robust image features from massive general datasets, which removed the requirement for big COVID-19 image databases [42]. ResNet50/101 and DenseNet121 architectures demonstrated superior accuracies compared with other CNN models [15,43]. ResNet models were the top-performing models in multiple investigations [5,12,20,22,44]. ResNet50 achieved the highest accuracy (98%) when Narin et al. (2021) tested various ImageNet CNNs on a CXR dataset containing 50 COVID-19 and 50 normal images [8]. Multiple research studies observed superior sensitivity and reliability of ResNet variant models when used for CXR and CT imaging tasks [20,22,24,25]. Hemdan et al. (2020) reported that VGG-16/19 and DenseNet-121 produced similar results, with both models achieving 90% accuracy when analyzing 50 CXR images [9]. The lightweight MobileNetV2 network demonstrated competitive accuracy levels exceeding 95% in larger examination scenarios and functions from a computational standpoint [5,30,45]. CNN-based classification models yield effective results with curated information, particularly when distinguishing normal lungs from COVID-19-infected lungs (Table 1). The number of training classes and the data diversity affect performance; however, data augmentation, together with fine-tuning and regularization techniques, supports accurate results [44].

### 4.2. Transformer-Based Models

Researchers have also examined vision transformers (ViTs) and attention-based models as alternatives to CNNs for diagnosing COVID-19 over the past two years [46]. The local connections within CNNs, called convolutional receptive fields, bias the network toward the difficult detection of the overall image context [47]. Transformers use self-attention protocols to process long-range relationships between elements, yielding high-performance outcomes when applied to vision tasks. The application of ViTs demonstrates their ability to process chest X-rays and CT slices [48]. The xViTCOS vision transformer model for COVID-19 screening using CXR/CT images was presented by Mondal et al. (2021) [49]. The attention heatmaps generated by xViTCOS during COVID-19 screening aid visual interpretation by marking infected regions through a multi-stage transfer learning method [50,51]. The Siamese vision transformer developed by Al Rahhal et al. (2022) operates in parallel with an image and its augmented version to achieve superior accuracy across CXR and CT datasets [52]. The transformer models demonstrated accuracy and sensitivity levels that matched the most effective CNN models and showed exceptional data efficiency when working with small training datasets [53]. Transformers generate attention maps that medical experts find easy to interpret because their focus area is concentrated on relevant lung regions, which improves trust in the model’s diagnostic capabilities. Data augmentation techniques, together with pretraining methods, make the use of ViTs with larger training sets better than CNNs in COVID-19 imaging applications [54,55]. Transformers and hybrid models that use CNN backbones with transformer blocks represent new developments in COVID-19 diagnostic AI, as they offer an opportunity to improve performance beyond the limitations of CNNs [56,57,58].

### 4.3. Ensembles and Hybrid Models

The varied performance of different architectures during model training has motivated experts to combine the use of multiple models to achieve higher accuracy levels. Different levels of ensemble learning may be implemented for information fusion, as explained in the subsequent section on information fusion methods. One standard method merges deep features acquired from several CNN models before classification by combining them into a more detailed representation [39,59]. Ilhan et al. (2021) extracted features from seven CNN models (including ResNet and VGG) for CXR images, which they consolidated into one vector to derive classification from multiple classifiers [16]. Combining different network models proved beneficial, as it delivered improved accuracy and detection efficiency compared with the use of isolated models, demonstrating the joint benefits of using multiple approaches. Decision-level fusion represents a technique that merges multiple model outputs using vote-counting processes or arithmetical combining methods. The same CXR ensemble used by Ilhan et al. achieved improved accuracy of more than 90% in three-class (COVID-19 vs. pneumonia vs. normal) analysis through their majority voting system, which surpassed the 88% accuracy of the best single classifier. Deep learning methods are currently combined with traditional machine learning approaches through hybrid solutions. According to Sethy & Behera (2020), combining a pre-trained CNN as a feature extractor, followed by an SVM training stage on developed features, produced 95% accuracy for X-ray images [60]. Various research studies have designed specialized CNN models for detecting COVID-19 in medical images [5,61]. The research community has gained access to the open-source COVID-Net, which has proven effective in detecting COVID-19 in CXRs, achieving an accuracy level of over 90%. Segmentation classification pipelines involving U-Net or UNet++ have been employed for CT analysis; they first divide the lungs into separate regions and then use a classifier CNN to determine the probability of the presence of COVID-19 in each segment [15,62,63]. These integrated frameworks focus on important elements and perform well (UNet++ had a 98.8% success rate using extensive CT datasets) [28,64,65]. The presence of COVID-19 is analyzed using diverse deep learning methods, including basic CNN classifiers, advanced ensemble structures, and transformer networks. The results of representative studies that evaluated CXR and CT data are presented in Table 1 and Table 2, respectively.

Several publicly accessible databases were used to compare the efficiency of convolutional neural networks (CNNs) and vision transformers (ViTs) in detecting COVID-19. These datasets differ in terms of imaging modalities, size, and diversity, which are essential aspects that contribute to the generalization of AI models [66]. Table 3 presents peer-reviewed articles that compare convolutional neural network (CNN) with vision transformer (ViT) models for COVID-19 detection in chest X-ray (CXR) and/or CT scan images. The information provided includes the names and availability of the datasets used, their diversity, and the key results (comparison of CNN vs. ViT performance).

These studies indicate that the ViT-based models can match the performance achieved by CNNs on COVID-19 chest images, or even exceed it when the availability of training data is high or when the models need to generalize to a different dataset (e.g., transfer to a different hospital) [73,74]. The diversity of the training data is very relevant to the generalizability of results; e.g., the enormous size of multi-source X-ray datasets (COVID-QU-Ex, COVIDx, etc.) enabled both CNNs and ViTs to achieve high accuracy; when trained on more limited and often homogeneous datasets, the global feature learning of transformers is useful for generalization [66,67].

## 5. Information Fusion Strategies for Enhanced Diagnosis

A key theme in recent research is how to fuse information from multiple sources or models to improve COVID-19 detection. “Information fusion” can occur at different levels in a deep learning pipeline:

### 5.1. Data-Level (Early) Fusion

Raw data from different modalities are combined as input to a single model [75]. The network framework receives simultaneous image and non-image data entries as part of its processing method. Data-level fusion in COVID-19 applications consists of linking different imaging views or modalities for analysis [76]. One system used a combination of CXR images together with blood test values as input by creating a stacked format, which merged clinical and image features prior to classification [3,77,78]. Wu et al. (2020) combined two orthogonal CT views using ResNet50 so that they could simultaneously fuse these views during input processing [22]. Joint data processing requires data modalities to appear simultaneously with proper normalization to achieve early fusion [78,79].

### 5.2. Feature-Level (Mid) Fusion

This technique merges different models and modalities into a unified representation. During multimodal system processing, separate subnetworks operate on different inputs, which is a common practice. Althenayan et al. (2024) combined COVID-19 diagnosis tests by linking separate CNN feature extractors for chest X-rays with tabular clinical data to distinguish COVID-19 from pneumonia [80,81]. Ilhan et al. (2021) combined seven CNN architecture deep features into a single vector representation for use in classification [16]. Combining various features at the attribute level yields superior results compared with separate features, as it provides expanded information input for the classification system. The combined feature vector faces a major limitation because it generates high-dimensional information, resulting in some studies implementing feature selection and dimensionality reduction. Ali et al. (2024) recently developed an approach featuring the extraction of deep features from multiple pre-trained networks, followed by feature selection through bio-inspired optimization components (Harris Hawks optimizer, along with particle swarm and others) to choose key feature subsets [82,83]. The optimized features achieved high accuracy when they were introduced into an SVM classifier after several networks combined their data points and eliminated unnecessary data points. The design automated the selection of features between pre-trained models to achieve an accurate 97.7% COVID-19 detection rate against other infections through X-ray analysis. The system demonstrates the benefits of accurately combining features to boost performance metrics.

### 5.3. Decision-Level (Late) Fusion

The output selections from multiple classifiers or models are combined during this process. The ensemble methods perform decision combination using different approaches, such as majority voting, weighted voting of prediction probabilities, or averaging methods. Through decision fusion, users can benefit from different output patterns since one model identifies instances that the others overlook. The detection of COVID-19 beneficially utilizes ensemble CNN models by allowing networks to vote for the final diagnosis [83]. The use of majority voting between Softmax and SVM algorithms using RBF and polynomial kernels led Ilhan et al. to improve overall CXR classification accuracy to 90.7% [16]. According to Frontiers’ survey results, an ensemble consisting of AlexNet, VGG-16, VGG-19, GoogLeNet, and SqueezeNet achieved 99.5% accuracy on CXR data while performing better than individual models [5]. Such late fusion techniques are easy to implement and generate stronger predictions because they offset the individual errors among models. The advantages of model fusion may be diminished when the ensemble consists of models that have similar errors or biases from data collection issues [84,85]. The implementation of these fusion strategies occurs in combination with one another during practical applications. Ilhan et al. (2021) delivered an effective pneumonia X-ray classification by applying both feature-level and decision-level fusion strategies, combining CNN features, and bringing multiple classifiers together [16]. Their approach achieved better precision/recall compared with using a single model as a baseline. The multimodal hierarchical model developed by Ali et al. (2024) combines image inputs and tabular features at an attribute level and uses a medical decision hierarchy to first classify healthy individuals versus patients with pneumonia before identifying pneumonia types [83]. Their approach of adding external clinical characteristics into a systematic decision framework enabled them to achieve an 87.5% F1-score on the eight-class identification of COVID-19 despite the task’s complexity. Using improved information fusion approaches, which combine various data types or model outputs, has proved effective in improving COVID-19 diagnosis accuracy [86,87]. Information fusion takes advantage of individual strengths and minimizes individual weaknesses from data sources, making it crucial in data environments with limited information quantity and distribution variability [88].

Feature-level fusion currently represents the primary and most robust fusion strategy for COVID-19 diagnosis due to the balance between taking advantage of rich, yet complementary data across modalities, maintaining reasonable complexity, and interpretability. Feature-level fusion uses complementary information, such as imaging to capture lung pathology and clinical data to capture systemic inflammation (CRP, oxygen levels, and age), and employs global context learning, particularly in combination with transformers or graph neural networks (Table 4). It also permits a fine-grained crossmodal view instead of merely accumulating end decisions [39,58,72].

As recent empirical studies on COVID-19 diagnosis have proven, a sensible staging of feature-level and decision-level integration is productive. The strengths of both paradigms are combined by first composing heterogeneous sets of features and then using ensemble decision-making strategies. The result of such hybridization is more accurate and consistent diagnoses, thereby reducing the inherent complexity of medical data. A notable example is the integration of deep feature extraction and ensemble classifiers, which was shown to be more effective than separate fusion algorithms and provides data about the patient at a deeper level [16,74].

## 6. Major Sources of Non-Uniformity in Chest Imaging Datasets for COVID-19 Detection and Their Impact on the Model

When applying DL for the detection of pneumonia caused by COVID-19 or other variants, it is very important that the input data adheres to established preprocessing and quality standards. Failure to ensure data consistency may lead to results that deviate from actual clinical outcomes. This can occur when scans collected from various hospitals are presented in different formats, when the data is ambiguous, or when relevant cases are omitted, introducing non-uniformities that may result in misleading or erroneous predictions (Table 5).

The impact on model generalization is presented in Table 6.

Not all COVID-19 detection models derived from imaging data are robust. This is due to non-uniformity, which has a direct effect on generalization, clinical applicability, and patient safety. Abating the sources of these non-uniformities is as important as the architecture of the models themselves [58,73].

## 7. The Role of Federated Learning in Ensuring Data Privacy and Enhancing Model Robustness in Healthcare

Federated learning (FL) is a process that allows multiple institutions to train machine learning models collaboratively, without exchanging any sensitive patient data [91]. Every institution maintains its data locally and only exchanges model updates (e.g., gradients or weights), which significantly reduces security concerns [92]. The approach adheres to data protection laws, such as GDPR and HIPAA, which play a significant role in the healthcare industry. By training on decentralized data (data from multiple healthcare providers), which include a greater diversity in patient demographics, imaging equipment, and disease manifestations, FL can capture a wider range of data [93]. Such variety enhances the model’s generalization property and makes it applicable in different clinical settings. The convergence of knowledge from federated learning overcomes the effects of non-uniformity in datasets by collecting information from diverse sources [94]. The result of this is that by using ensembles, model performance is balanced in a more unbiased manner, even if each institution has skewed or biased data. FL reduces the need for central massive storage and infrastructure configurations. Institutions also help with training models using local computational resources, thus enhancing scalability without compromising data sovereignty [95]. Training in federated learning enables the application of methods such as secure multiparty computation and differential privacy [96]. These procedures also guard against the leaking of sensitive information during model updates such that sensitive information cannot be de-engineered [97]. In cases where rare diseases or conditions are present, federated learning enables data to be kept segregated, but knowledge is shared. Such group knowledge enhances model performance on rare conditions, which would be challenging to achieve using data from a single institution. Federated and distributed learning is another paradigm shift in AI in healthcare. They also ensure the privacy of the patients and improve model quality between institutionalization. These methods democratize access to collective intelligence without compromising privacy and create a path towards more equitable, accurate, and scalable AI-powered solutions in healthcare.

## 8. Evaluating Model Resilience to Image Artifacts, Comorbidities, and COVID-19 Mimickers

Artifacts such as noise, poor contrast, motion blur, and hardware-based distortions are frequent in images in clinical settings. Such artifacts may cover important characteristics, preventing accurate diagnosis. Models that have been primarily trained on clean and curated datasets may struggle to handle artifact-ridden images and labels, leading to reduced sensitivity and specificity, particularly for subtle COVID-19 cases (Table 7). Resilience can be improved by using techniques such as data augmentation (e.g., adding noise and blurring), adversarial training, and artifact-detecting models, etc. [98]. Attention mechanisms are artifact-resistant architectures that are sometimes included to focus on undamaged parts of the image. COVID-19 often occurs concurrently with diseases such as chronic obstructive pulmonary disease (COPD), pulmonary edema, or cancer. These comorbidities cause an overlap in the patterns observed during radiology (e.g., ground-glass opacities and consolidations). Deep learning models can confuse COVID-19 with underlying diseases, unless they have access to extensive training examples [99]. This leads to inaccurate positive or negative conclusions, resulting in clinically unreliable results. Model generalization is enhanced by the inclusion of complete datasets that depict a variety of comorbid conditions. Some models also employ multimodal architectures, integrating imaging with non-imaging information (e.g., laboratory results, patient history, etc.) to discriminate COVID-19 from other diseases [25]. Bacterial and other viral infections (e.g., influenza, SARS, and MERS) that cause pneumonia result in images similar to those of COVID-19, including bilateral opacities or consolidations. Without differentiation training, high rates of model misclassification can occur [58]. Although it is essential to distinguish COVID-19 from other infections, this task is difficult, as the radiological variations are minimal. Contrastive learning, transfer learning across similar domains, or ensemble-based models are superior in discriminating between COVID-19 and other respiratory infections [70]. The application of annotated datasets with de facto labels of different types of pneumonia enhances diagnostic specificity. Differences in the resilience of AI models used for COVID-19 diagnosis can be observed due to the varying degrees to which they were trained to account for artifacts, comorbidities, and other respiratory infections [72]. Although aspects such as data diversity, data augmentation, and multimodal learning have contributed significantly to highly robust modeling, other models are still weak to the variability that exists in nature [89]. The current areas of research emphasize the significance of the quality and diversity of the dataset, as well as the need to elaborate on model architecture to increase resilience for clinical deployment.

The resilience level and factor are presented in Table 8.

## 9. The Role and Importance of Explainable AI (XAI) in Clinical Diagnosis

Explainable AI (XAI) is proving to be a critical element in the implementation of machine learning models, especially in sensitive areas such as healthcare. The use of XAI in clinical AI applications, such as classifiers, detectors, and decision-support tools, has numerous benefits from ethical, scientific, and practical standpoints [100].

(*i*) Ethics and Laws

For healthcare decisions involving AI to be fair, transparent, and accountable, they should be interpretable and explainable. XAI enables clinicians and regulators to understand the reasoning underlying AI-generated predictions or diagnoses [100]. The General Data Protection Regulation (GDPR) is one example of a legislation that implements the so-called right to explanation—the requirement to provide an explanation for any automated decision that affects an individual. XAI is compliant with this requirement, allowing AI systems to be launched in controlled clinical practices. XAI can also help identify cases where a model is relying on outdated or biased variables (e.g., demographic characteristics), which can prevent discrimination and promote equality in healthcare [101].

(*ii*) Scientific Validity and Concordance with Medical Sources

XAI techniques (e.g., saliency maps, Grad-CAM, and SHAP) enable clinicians to visualize the areas of a chest X-ray or CT scan that most significantly influenced the model’s prediction. Such conformance with current pathological findings (e.g., ground-glass opacities in COVID-19) increases the validity of AI results [102]. It is possible to cross-validate models with the existing clinical literature. For example, when an AI model uses lung peripheries to diagnose COVID-19, this aligns with the clinical knowledge of the radiological appearance of the disease. XAI traces model failures, which can help clinicians and developers improve the diagnosis process, minimizing the dangers associated with false predictions [103].

(*iii*) Confidence, Adoption, and User Acceptance

Clinicians will be more willing to use AI tools when they understand how these models reach decisions. Transparent models mitigate the black box effect, fostering trust in the technology. XAI tools also serve as a teaching tool for less experienced clinicians, offering explanations about minor radiologic patterns or a complicated decision path [104]. Explainability can help clinicians leverage AI in an advisory capacity, rather than as a substitute; this can improve the accuracy of diagnoses, since the decision-making process will be conducted by both sides. When explainable AI methods are used within clinical models, they address ethical and legal issues, align AI outputs with clinical texts, and provide trust and acceptance among care providers [105]. Consequently, XAI appears to be not only a technical improvement but also the cornerstone of the safe development and effective and ethical implementation of AI in clinical practice.

## 10. Key Challenges and Limitations

Despite rapid progress, deep learning-based COVID-19 detection faces several challenges:

Limited and Imbalanced Data: Publicly available COVID-19 imaging data remain limited compared with those of other diseases (Table 9).

A sufficient number of COVID-19 cases were obtained from several imaging datasets [10,13,18,19,22,30,39,62]. COVID-19 detection models tend to make errors due to their preference for the majority classification, resulting from an uneven distribution between COVID-19 and normal/pneumonia image data. The success rate of synthetic data augmentation strategies that combine flips and rotations with GAN-generated images has been reported in addressing this issue [72]. Small, non-diverse datasets do not work well for model training because they impair the models’ generalization ability. Early studies reported exceptionally high accuracy results (typically 95–100%); however, these measurements were likely inaccurate because test samples were used during training stages [5]. Thus, research focused on performing cross-dataset evaluations and using more authentic benchmarking standards.

**Highlights:** 

- Top accuracy: Loey et al. (100%) [37]
- Top sensitivity: Ardakani et al. (100%) [30]; Ni et al. (100%) [112]
- Largest dataset: Ni et al. (14,531 images) [112]

### 10.1. Data Quality and Noise

Hospital medical imagers utilize different parameter settings during data acquisition. Intensity distribution shifts, known as the multi-domain issue, occur due to differences between CT scanners [116]. The quality of CXRs depends on the technological differences between portable bedside X-rays and departmental X-rays. Several COVID-19 datasets were hurriedly developed and contain both tagging inconsistencies and poor-quality images [5]. A model’s accuracy can be reduced when medical practitioners label COVID-19 patients as having pneumonia or when labeling errors, such as patient identity tags, exist in training images. The construction of data platforms requires extensive attention and should include training approaches that can tolerate label faults during modeling. Two approaches—weakly supervised learning and domain adaptation (e.g., the CIFD-Net model)—address these problems by developing domain-invariant features and uncertain label tolerance mechanisms [117,118].

### 10.2. Overfitting and Generalization

A recurring concern is that models might overfit to dataset-specific artifacts. For example, a model might learn to identify a certain hospital’s CT scans rather than the pathology. The difficulty in “singling out one best system” noted in surveys stems from the fact that each model is usually tested on a unique dataset [5]. When evaluated on a new dataset, performance often drops. This highlights the need for external validation. Some recent studies tested models on data from different hospitals or countries and found noticeable performance degradation, highlighting the generalization challenge [119,120]. Ensemble and fusion methods help by making models more robust; however, they cannot fully resolve the issue if the training data themselves are not representative [121].

### 10.3. Evaluation Metrics and Reporting

Medical diagnosis tasks require sensitivity and specificity metrics instead of accuracy-based measurements because they offer better diagnostic accuracy. A high overall accuracy value can be untrustworthy when the training data contains an uneven distribution of categories. The model achieves high accuracy through correct non-COVID-19 case identification and total COVID-19 case misclassification, which makes it unusable in medical practice. Sensitivity (recall), specificity, precision, the F1-score, and AUC constitute the modern set of metrics used for assessing COVID-19 diagnostic systems [5,122,123]. Sensitivity takes precedence because undetected cases pose serious public health risks [124]. A large number of models demonstrate sensitivity rates ranging from 95% to 100%, although they often trade off specificity as a result [125,126,127]. The detection of positive cases requires strict threshold tuning because it remains difficult to achieve balanced metrics across these measures. The comparison of different models has become complex because some researchers employ cross-validation while others choose train/test splits as their evaluation protocols [128,129].

### 10.4. Clinical Integration and Trust

Implementing accurate models into clinical processes can be challenging, even if their predictions demonstrate high accuracy. Healthcare providers need detailed justification for AI predictions during critical assessments of patient outcomes. Research on explainable AI has resulted in the development of saliency maps, which show areas in lung images that affect prediction results [49]. Using the model xViTCOS, users can detect both predictions and their linked abnormality locations. AI systems require the accurate management of real-world prevalence data because changing COVID-19 infection rates modify the accuracy measurements of predicted diagnoses [130]. A learning system optimized through high prevalence training generates additional false positives if deployed in an environment with low prevalence [131]. AI systems require formal regulatory assessments and multi-center trials for validation prior to their deployment. Regulatory barriers currently limit the approval of COVID-19 AI diagnostic tools, mainly because of technological challenges. The extensive research on the pandemic has led to the development of better methods for creating AI systems quickly, despite the limited availability of data.

## 11. Recent Advancements and Future Directions

Research on deep learning for COVID-19 diagnosis conducted from 2020 to 2024 has led to notable advancements while also creating new avenues for future work:

### 11.1. Improved Model Performance

Large international collaboration efforts have resulted in the assembly of large datasets comprising thousands of COVID-19 cases, thereby enhancing model accuracy. The best models now achieve high (90%) sensitivity and specificity on test sets, and some ensembles approach the performance required for clinical use [5,132,133,134].

Developers have applied multi-task learning methods to train systems for diagnosing both COVID-19 severity and detecting areas of infection, thereby improving general clinical utility [106]. Modern vision transformers and hybrid CNN–transformer models represent recent developments that enhance both accuracy levels and model interpretability [135,136]. These models used vast chest X-ray databases (NIH ChestX-ray8 or CheXpert) for pre-training early in the pandemic; their applications were then fine-tuned to COVID-19 diagnosis [62,137,138,139].

### 11.2. Multimodal and Multi-Task Fusion

Research shows great potential in modality integration, which involves combining various data sources, such as clinical data, with imaging or computerized chest X-rays and computed tomography. The use of deep learning through various input methods enables medical professionals to gain an improved overview of patient health [140,141]. Imaging data, combined with patient vital sign measurements and laboratory results, enables models to distinguish COVID-19 from other diseases that generate analogous imaging results but display contrasting lab results [3]. Recent studies have demonstrated the ability to use time-dependent data by assessing image or vital sequence changes for COVID-19 diagnosis and monitoring progression or recovery [142]. The optimization of information fusion strategies may be accelerated through automated methods, such as the network/feature selection optimization by Ali et al. [83]. Future AI diagnostic systems will combine multiple inputs to generate medical diagnoses, along with their confidence levels and explanations.

### 11.3. Hierarchical and Explainable Models

Clinical decision tree structures form an emerging modeling approach for classification systems [72]. Model decisions begin with determining whether an image contains normal or abnormal findings, before proceeding to further class definitions. Following the identification of an abnormality, the algorithm proceeds to determine the pneumonia status, which leads to the identification of either COVID-19 or another type of pneumonia. Using this method, practitioners can achieve better results on unbalanced datasets while producing detailed diagnostic outputs, such as “COVID-19 pneumonia”, “non-COVID pneumonia”, or “no pneumonia” [72,97,104]. The use of this practice matches medical reasoning capabilities and works together with attention systems to target the right lung zones for data processing [143,144]. Explainability requires constant attention in medical operations; thus, experts are developing techniques such as class activation maps and attention maps in transformers, as well as prototype-based reasoning, to make AI decisions more transparent. Model transparency will be essential to obtain professional endorsement. Recent transformer models have demonstrated the ability to focus on ground-glass opacity regions, which matches radiologists’ intuitive observations.

### 11.4. Generalization and Deployment

Generalization problems in healthcare have inspired researchers to develop federated learning frameworks that train on distributed hospital data while protecting patient privacy [145]. The new approach enables the development of models that can handle various healthcare environments. Models should incorporate continuous learning frameworks to enable them to receive updates through new data entries, such as new variants and populations, thereby extending the lifespan of the tools. Experts have aimed to extend the application of these detection systems from diagnostic purposes to different functions, such as the assessment of critical patient populations [146]. Research studies have progressed from detecting COVID-19 to making prognostic (outcome prediction) evaluations using deep learning approaches [147]. Deep learning coupled with information fusion demonstrates quick adaptability in new diagnostic challenges, and this method is expected to be effective for other emerging diseases or future pandemic detection [15,146].

## 12. Conclusions

Deep learning has proven to be a formidable tool for COVID-19 detection, enabling the rapid diagnosis of the disease from chest images and the assessment of medical data. The detection of COVID-19 is facilitated by numerous neural network designs, including traditional CNN-based approaches and modern transformer models, which achieve superior results on evaluation sets. Network selection optimization and information fusion approaches have significantly improved accuracy by merging multiple models and data types. The studies investigated in this review indicate that since 2023, COVID-19 detection using AI has achieved accuracy rates of over 90%, a level that, in some cases, matches that of expert radiologists. The medical application of current advancements needs to resolve problems associated with data quality, as well as issues concerning generalizability and trust. COVID-19 detection now involves multiple large-scale testing centers and clearer diagnostic methods and models, which offer comprehensive patient assessments. The application of deep learning in COVID-19 detection has witnessed significant improvements in the past few years, strengthening conditions for AI-based diagnostic platforms. Mechanical advancements in model strengthening, data integration, and human–AI network collaborations will enhance our preparedness for current and future healthcare crises.

## Figures and Tables

**Table 1:** Representative deep learning models for COVID-19 detection on chest X-ray images. Each study uses different class setups (binary vs. multi-class) and evaluation protocols; therefore, performance metrics are not directly comparable.

Study (Year)	Data (Chest X-Ray)	Classes/Task	Model(s) and Approach	Performance
Narin et al. (2021) [8]	100 images (50 COVID and 50 normal)	COVID vs. Normal (binary)	ResNet50 (TL fine-tuned)	Acc 98.0%
Hemdan et al. (2020) [9]	50 images (25 COVID and 25 normal)	COVID vs. Normal (binary)	VGG19 and DenseNet201 (TL; ensemble reported)	Acc~90%; F1 91%
Apostolopoulos et al. (2020) [10]	~2900 images (mixed sources)	COVID vs. Pneumonia vs. Normal	MobileNetV2 (TL)	Acc 96–98% (3-class)
Horry et al. (2020) [11]	400 images (100 COVID, 100 pneumonia, and 200 healthy)	COVID vs. Pneumonia vs. Normal	Inception, Xception, ResNet, and VGG (ensemble)	Prec 83%; Sens 80%; F1 80%
Bukhari et al. (2020) [12]	278 images (3 classes: COVID, normal, and pneumonia)	COVID vs. Pneumonia vs. Normal	ResNet50 (TL fine-tuned)	Acc 98.2%; Sens 98.2%; F1 98.2%
Benmalek et al. (2021 [13]	158 images (COVID vs. others)	COVID vs. Other diseases (binary)	ResNet50 + SVM (a CNN for features and an SVM classifier)	Acc 95.4%
Minaee et al. (2020) [14]	5071 images (many sources; 100 COVID)	COVID vs. Normal (binary)	Ensemble of ResNet18, ResNet50 and SqueezeNet (TL)	Acc 98.0%; Sens 100%
Kaya & Gürsoy (2023 [15]	9457 images (3-class dataset)	COVID vs. Pneumonia vs. Normal	MobileNetV2 (TL; 5-fold cross-val)	Acc 97.6% (3-class)
Ilhan et al. (2021) [16]	~23,000 images (from 3 public sets combined)	COVID vs. Pneumonia vs. Normal	Feature fusion of 7 CNNs + ensemble classifiers	Acc 90.7%; Prec 93%; Sens 91% (3-class)

**Table 2:** Representative deep learning models for COVID-19 detection from chest CT scans.

Study (Year)	Data (Chest CT)	Classes/Task	Model(s) and Approach	Performance
Wu et al. (2020) [22]	495 CT images (368 COVID and 127 other infections)	COVID vs. Other infections	ResNet50 (TL and multi-view fusion of slices)	Acc 76%; Sens 81%; Spec 62%
Xu et al. (2020) [23]	– (CT images and a small dataset)	COVID vs. Non-COVID (binary)	ResNet18 (TL, with lung segmentation)	Acc 86.7%
Rehman et al. (2022 [24]	– (CT and a three-class problem)	COVID vs. Viral vs. Bacterial Pneu.	ResNet101 (TL fine-tuned)	Acc 98.75% (on the COVID class)
Jin et al. (2020) [25]	1881 images (496 COVID and 1385 healthy)	COVID vs. Normal (binary)	ResNet152 (TL fine-tuned)	Acc 94.98%
Afshar et al. (2022 [26]	1020 CT scans (COVID vs. healthy)	COVID vs. Normal (binary)	ResNet101 (TL; evaluated on the hold-out set)	Acc 99.5%
Yousefzadeh et al. (2021) [27]	2124 CT scans (706 COVID and 1418 normal)	COVID vs. Normal (binary)	Ensemble of ResNet, EfficientNet, DenseNet, etc. (COVID-AI)	Acc 96.4%
Chen et al. (2020) [28]	46,096 CT images (from hospital)	COVID vs. Normal (binary)	UNet++ + ResNet50 (segmentation + classification)	Acc 98.85%
Javaheri et al. (2021) [29]	89,145 CT images (32 k COVID, 25 k CAP, and 31 k healthy)	COVID vs. CAP vs. Normal (3-class)	CovidCTNet (3D U-Net for localization + CNN)	Acc 91.66%; Sens 87.5%; Spec 94.0%; AUC 0.95

**Table 3:** Key studies comparing CNNs vs. vision transformers for COVID-19 chest imaging.

Study (Year)	Datasets (Modality)	Public Availability	Dataset Diversity	Key Findings (CNN vs. ViT)
Nafisah et al., 2023 [66] *MDPI Mathematics*	COVID-QU-Ex (CXR): ~21,165 images (10,192 normal, 7357 non-COVID-19 pneumonia, and 3616 COVID-19) with lung masks [66,67].	Yes—open dataset (COVID-QU-Ex) available via Kaggle	Multi-source and large: Compiled from multiple public repositories (the largest COVID-19 CXR dataset). Images vary in quality, resolution, and source (frontal CXRs from diverse hospitals), enhancing generalizability [67].	CNN vs. ViT: Achieved *comparably* high accuracy. The best CNN (EfficientNet-B7) achieved 99.82% accuracy, slightly outperforming the best ViT model (SegFormer, 99.7% range) [66]. Both model types showed near-ceiling performance on this dataset, particularly after lung-region segmentation and augmentation, indicating no clear advantage of the ViT over the CNN in this setting.
Ferraz & Betini, 2025 [68] *J. Brazilian Comp. Society*	COVID-QU-Ex (CXR), HCV-UFPR-COVID-19 (CXR), HUST-19 (CT), and SARS-CoV-2 CT-Scan (CT). These four benchmark datasets cover both X-rays and CTs [67,68].	Mixed: COVID-QU-Ex, HUST-19, and SARS-CoV-2 CT are public; HCV-UFPR is private (available upon request). COVID-QU-Ex and HUST-19 are open-access datasets [68]; the HCV-UFPR X-rays are provided on a case-by-case basis by Hospital da Cruz Vermelha (Brazil) [69]; the SARS-CoV-2 CT set is from Kaggle (Al Rahhal et al., 2022) [52].	COVID-QU-Ex: Multi-institution global CXR collection (highly diverse in source institutions and imaging conditions) [70]. HCV-UFPR: Single-hospital in Brazil (281 COVID-19 and 232 normal CXRs [69]; limited demographic variety). HUST-19: Large CT dataset (≈13,980 images from ~1521 patients) from Huazhong Univ. hospitals in Wuhan [65]—sizable but geographically localized. SARS-CoV-2 CT: Collected from multiple hospitals in São Paulo (2482 CT images) [71]; some diversity within one region.	CNN vs. ViT: All models achieved strong classification results, but the Swin Transformer (ViT) consistently outperformed the CNN (ResNet-50) on both CXR and CT tasks [67]. Notably, Swin demonstrated greater generalization in cross-dataset experiments (training on one dataset and testing on another), achieving an AUC/accuracy of up to 1.0 on some test sets [67]. This suggests that ViT-based models (especially Swin) handle distribution shifts between institutions better than the CNN in this study.
Padmavathi & Ganesan, 2025 [72] *Scientific Reports (Nature)*	COVID-QU-Ex (CXR, 33,920 images across COVID-19/non-COVID-19/normal classes, with ground-truth lung masks) and a Wuhan CT Collection (CT images from Union and Liyuan Hospitals, Wuhan; 8000 CT slices balanced between COVID-19-positive and normal) [72].	Yes—both datasets were made public by the authors on Kaggle (the CXR set is the COVID-QU-Ex Kaggle version, and the CT set contained preprocessed data from the two hospitals) [72].	COVID-QU-Ex: Very diverse; drawn from multiple international CXR sources; covers varied patient demographics, imaging devices, and conditions [67]. Wuhan CT dataset: Collected from two large hospitals (common imaging protocols); less geographic diversity (all patients from the same region), but a substantial sample size, ensuring statistical power [72].	CNN vs. ViT: A ViT-based approach with optimization techniques significantly outperformed classic CNNs in COVID detection. The proposed hybrid ViT model achieved ~99.1% accuracy in binary CXR classification, compared with 84–93% for standard CNN baselines (ResNet34, ~84.2%; VGG19, ~93.2%) [72]. Similar trends were observed with the CT data (~98.9% vs. mid-80 s%). The ViT’s attention mechanism (enhanced by Gray Wolf and PSO optimizers) captured global lung features, yielding superior performance and robustness [72]. This highlights significant performance gains for ViTs over CNNs in both modalities when advanced tuning is applied.
Tehrani *et al*., 2023 [58] *BMC Med. Inf. and Dec. Making*	Private Iran COVID-CT Dataset: 380 COVID-19 patients’ CT scans (each with 50–70 slice images) plus corresponding clinical data (demographics, vitals, labs) [58]. After preprocessing, 321 patients’ data were used for outcome prediction (e.g., survival vs. deterioration) [58].	No (Private)—Data were collected in-house and are not publicly available (only shared by authors upon reasonable request) [58].	Locally collected: All CT scans and patient records come from a limited number of hospitals (single country); thus, patient demographics and scan conditions are relatively homogeneous. The authors note the challenge of obtaining large multi-center clinical datasets [58]. (They mitigated data scarcity using data fusion and augmentation, but the dataset lacks the multi-national diversity of open datasets.)	CNN vs. ViT: This study fused 3D chest CT images with clinical features to predict disease outcomes. A 3D video Swin transformer (ViT) model outperformed several CNN models, given the same input data [58]. In predicting high-risk patients, the Swin transformer achieved the highest true-positive rate (~0.95) and best overall AUC (0.77) among the tested models [58]. In contrast, conventional 3D CNNs on the same task showed lower accuracy (The TPR for CNNs was lower, not reaching 0.95). This indicates the ViT’s stronger ability to leverage 3D imaging + clinical information for COVID-19 severity prediction.

**Table 4:** Comparison of fusion strategies.

Fusion Type	Description	Performance Summary	Strengths	Limitations
Data-Level Fusion	Merges aggregate data (or images) across several modalities (e.g., CT + X-ray images or image + clinical data) into a common input space.	Imaging + clinical data are rarely used. They are used in imaging (e.g., multi-view X-rays). Middling gains are easily susceptible to noise gain.	Stores the largest quantity of data; easy to apply to data of the same type (e.g., multi-view X-rays).	Heavy computational time requirements; high dimensionality explosion of inputs; sensitivity to missing data; concept drift; modality alignment task.
Feature-Level Fusion	Performs fusion on latent features produced by modalities (e.g., picture embeddings + clinical) prior to the last prediction levels.	Highest performance in most COVID-19 studies. Other models, such as transformer-based designs with concatenated image + clinical features, are superior.	Balances complexity that can be handled and the richness of information. Allows each mode encoder to become specialized. Resistant to noise; resistant to non- homogeneous data (CT + lab data).	Mandates scrupulous architectural design of fusion layers to align the dimensions and semantics of features.
Decision-Level Fusion	The modules of each modality are separated; the outputs (labels or probability scores) of each model are brought together through either voting, averaging, or meta-learners.	Performs well on small datasets or in heterogeneous modalities. Provides robustness at the cost of usually not being as accurate as feature-level fusion when abundant data are available	Practical to apply; independent modes minimized cross-modality interference; tolerant to failure in one of the modes.	Overlooks the interaction of deep features across modalities; has a lower performance limit than feature fusion.

**Table 5:** Major sources of non-uniformity [89,90].

Source of Non-Uniformity	Description	Examples from COVID-19 Datasets
Device and Scanner Variability	Variability across X-ray or CT machines (brand, model, imaging resolution, and imaging protocol).	- The COVIDx dataset contains scans from several hospitals, generated using different machines. - COVID-QU-Ex combines images from various equipment, which have various image qualities.
Acquisition Protocol Differences	Variation in patient positioning (AP vs. PA view in X-rays), slice thickness in CT, exposure time, and contrast use.	- Other datasets are a combination of AP and PA chest X-rays, which have not been standardized. - CT data differ in slice thickness (1 mm vs. 5 mm), which affects the level of detail observed in the lung images.
Patient Demographics	Differences in age, sex, ethnicity, geography, and comorbidities.	- COVID-QU-Ex has a worldwide distribution, but it lacks representation of some ethnicities. - The HUST-19 CT dataset has less diversity, as it primarily contains data from individuals from Wuhan.
Annotation Inconsistency	Lip antenna variation in labels because of divergent standards of diagnosis, manual vs. automated labeling, or mistakes.	- COVIDx contains labels derived from text reports as opposed to radiologist consensus. - Other CT datasets have slices, which are ambiguous, instead of patient labels.
Image Preprocessing Differences	Differences in normalization, resizing, windowing (particularly of CT), and cropping of images.	- Differing methods of normalizing CT scans. Some datasets normalize to [−1000, 400 HU], while others normalize to [−1024, 3071 HU]. - In certain datasets, chest X-rays can be cropped to lung areas, while there is no such cropping in other datasets.
Class Imbalance and Selection Bias	Artificially high proportions of COVID-19-positive cases or other conditions; selection within particular hospital groups.	- COVID-19 data are biased towards severe (hospitalized patients) vs. mild/asymptomatic cases. - It is possible that some datasets either excluded normal cases or included more pneumonia controls in populations.

**Table 6:** Impact on model generalization [89,90].

Source	Impact
Device Variability	There is a risk that models are scanner-dependent and modules learn scanner patterns (gridlines and noise patterns) instead of pathology. Inefficient when used on various scanners in the hospital.
Acquisition Protocol Differences	Inconsistent characteristics on account of angles of projection (e.g., AP vs. PA), which results in a loss of accuracy when dependencies between deployment data are not the same in the methods of acquisition. Example: Models based on the frontal view may misdiagnose lateral images.
Patient Demographics	Poor generalization to unobservable populations (e.g., age groups and ethnicities). A model trained on a majority of elderly patient cases may not work on pediatric patients or young adults.
Annotation Inconsistency	Labeling errors are a source of noise in training data, resulting in an inflated level of false positives/negatives. Disadvantages: It decreases the maximum accuracy that can be attained and compromises reliability.
Preprocessing Variability	Models trained to perform on a specific preprocessing pipeline (e.g., cropped lungs) fail when test images have gone through a different preprocessing pipeline. It is particularly useful in the context of transfer learning and deployment.
Class Imbalance	Prejudice against overrepresented classes (e.g., overrepresentation of COVID-19-positive samples in a dataset where COVID-19 cases represent 80 percent of the data, whereas in the real world, it is only ~10 percent) causes inaccuracy in real-life screening.

**Table 7:** Key challenges to model robustness.

Challenge Type	Description	Example Confounders
Image Artifacts	The non-biological characteristics that mask or resemble an image.	Motion blur, metal implants, ECG leads, portable X-ray artifacts, and under/overexposure.
Comorbidities	Other systemic or pulmonary diseases that can change the imaging findings.	COPD, pulmonary fibrosis, lung cancer, and heart failure (causing pulmonary edema).
Other Respiratory Infections	Non-COVID-19 pneumonias or viral infections that share imaging features.	Bacterial pneumonia, influenza, SARS, MERS, and tuberculosis.

**Table 8:** Summary of resilience performance.

Factor	Resilience Level	Observations from the Literature
Image Artifacts	Moderate to poor	-CNNs are highly sensitive to artifacts. Studies show that models often misclassify based on scanner noise, image borders, or embedded texts rather than lung pathology. -Vision transformers (ViTs) exhibit better resilience due to global context awareness but still degrade with severe motion blur or low-dose CT noise. -Data augmentation with synthetic artifacts improves robustness.
Comorbidities	Low to moderate	-AI models trained on COVID-19 datasets often fail to generalize to patients with overlapping conditions, such as heart failure or COPD. -Studies (e.g., Cohen et al., 2020 [89]) have found a high false-positive rate in elderly patients with comorbid pulmonary edema, as fluid accumulation mimics COVID-19 consolidations. -Feature-level fusion with clinical data improves performance (e.g., distinguishing COVID-19 from heart failure when oxygen saturation and BNP levels are included).
Other Respiratory Infections	Variable—Poor if not explicitly trained	-Differentiating between COVID-19 and non-COVID-19 pneumonia is the most challenging aspect in imaging-based diagnosis, since patterns such as ground-glass opacities are not COVID-19-specific. -Models trained without diverse non-COVID-19 pneumonia samples often show inflated accuracy, failing real-world deployment. -Well-curated datasets with balanced pneumonia classes (e.g., COVID-QU-Ex and COVIDx) improve resilience, but accuracy drops by 10–15% compared with COVID-19 vs. healthy tasks.

**Table 9:** Ranking table based on the average performance (accuracy, sensitivity, and specificity) of different studies.

Rank	Authors	Population	Technique	Model	Imaging Type	Key Results
1	Loey et al. [37]	306	DL	GoogleNet	X-ray	Acc 100%
2	Ko et al. [106]	3993	DL	ResNet-50 (FCONet)	CT Scan	Acc 99.87%, Sens 99.58%, Spec 100%, and
3	Hasan et al. [107]	321	TL	LSTM Classifier	CT Scan	Acc 99.68%
4	Ardakani et al. [30]	194	DL	AlexNet, VGG-16, VGG-19, GoogleNet, and SqueezeNet	CT Scan	Acc 99.51%, Sen 100%, and Spec 99.02%
5	Apostolopoulos and Mpesiana [10,108]	455	CoroNet (DL-based)	MobileNetV2	X-ray	Acc 99.18%, Sens 97.36%, and Spec 99.42%
6	Waheed et al. [108]	1124	GAN (CovidGAN)	ACGAN3 and VGG-16	X-ray	Acc 95%, Sens 90%, and Spec 97%
7	Rahimzadeh and Attar [39]	11,302	DL	ResNet50V2 + Xception	X-ray	Acc 95.5%, and overall 91.4%
8	Saiz and Barandiaran	1500	CNN + TL	VGG-16 (Single-Depth Dilation)	X-ray	Acc 94.92%, Sens 94.92%, Spec 92%, and F1-score 97
9	Wang et al. [62]	181	DL	VGG-19	X-ray	Acc 96.3%
10	Brunese et al. [109]	6523	CoroNet (DL-based)	VGG-16	X-ray	Acc 96.3%
11	Abbas et al. [110]	6523	CoroNet (DL-based)	VGG-16	X-ray	Acc 97%
12	Panwar et al. [111]	337	DL	VGG-16	X-ray	Acc 88.10%, Sens 97.62%, and Spec 85.7%
13	Ni et al. [112]	14,531	DL	3D U-Net + MVPNet	CT Scan	Sens 100% and lobe lesion score 0.96 (no Acc mentioned)
14	Pathak et al. [113]	852	TL	ResNet-50	CT Scan	Acc 93%
15	Yang et al. [19,114]	295	DL	DenseNet	CT Scan	Acc 92%, Sens 97%, and Spec 7% (very low specificity)
16	Pereira et al. [115]	1144	CNN	Inception-V3	X-ray	F1-score: 89 (no Acc reported)
17	Sethy et al. [60]	381	CNN + SVM	ResNet-50	X-ray	Sens 95.33% (no Acc mentioned)
18	Wang et al. [17]	5372	DL	DenseNet121-FPN	CT Scan	Acc 87–88% and Sens 76.3–81.1%
19	Wu et al. [22]	495	CoroNet (DL-based)	VGG-19	CT Scan	Acc 76%, Sens 81.1%, and Spec 61.15%

## Data Availability

Data sharing is not applicable.

## Abbreviations

The following abbreviations are used in this manuscript:

AI	Artificial intelligence
CNN	Convolutional neural network
COPD	Chronic obstructive pulmonary disease
CT	Computed tomography
CXR	Chest X-ray
DL	Deep Learning
FL	Federated learning
GDPR	General Data Protection Regulation
RT-PCR	Real-time polymerase chain reaction
ViT	Vision transformer
XAI	Explainable AI
X-Ray	Radiography
